# Analysis of the Accuracy of CAM-type Deformity Resection on a Low-cost Arthroscopic Simulator in a Training Scenario

**DOI:** 10.1055/s-0044-1785666

**Published:** 2024-06-22

**Authors:** Bruno Gonçalves Schroder e Souza, Vitor Homero Vieira, Marcos Miranda, Luiz Guilherme Vidal Assad de Carvalho, Flavia de Souza Bastos, João Vitor Delgado Vilas Boas

**Affiliations:** 1 Faculdade de Ciências Médicas e da Saúde de Juiz de Fora, Juiz de Fora, MG, Brasil; 2 Universidade Federal de Juiz de Fora, Juiz de Fora, MG, Brasil; 3 Cirurgia do Quadril, Hopspital Belo Horizonte, Belo Horizonte, MG, Brasil; 4 Departamento de Mecânica Aplicada e Computacional, Programa de Pós-Graduação em Modelagem Computacional, Juiz de Fora, MG, Brasil

**Keywords:** arthroscopy, femoracetabular impingement, hip, simulation training

## Abstract

**Objective** To evaluate surgeons' performance in resecting CAM-type deformities using a realistic arthroscopic surgery simulator.

**Methods** An arthroscopic simulator was created using low-cost materials with the help of a GTMax Core A1 3D printer and the programs Invesalius and Meshmixer 2017, which were used to develop femoral head parts in ABS material, with the presence of a CAM-type deformity, to mimic a femoroacetabular impact situation. After the operations were performed by 16 surgeons, the femurs were compared to a previous model with deformity and another without, using Cloudcompare, and parameters such as the volumetric difference between the operated femurs, with and without deformity, the minimum and maximum distance between them, the percentage of the deformity resected, the estimated time for total resection of the deformity, as well as a qualitative analysis based on the images and graphs provided by the program representing the areas of the parts resected, were evaluated at the end.

**Results** The average resection speed was 34.66 mm ^3^ /min (SD = 46 mm ^3^ /min, max = 147.33; min = -2.66). The average resection rate was 26.2% (SD = 34.7%, max = 111; min = -2). Qualitative analysis showed hyporesection of deformities and sometimes hyperresection of nondeformed areas. The simulator was highly rated by the surgeons, with a tactile sensation very similar to real surgery, according to them.

**Conclusion** Arthroscopic simulators have proved very useful in training less experienced surgeons.

## Introduction

Training surgeons to acquire complex motor skills is a decisive factor in the outcome of orthopedic surgery. A greater volume of cases has been strongly associated with improved post-operative recovery time, lower mortality, and lower costs in hospital settings. 1 The volume of training increases surgeons' efficiency in the operating room and patient safety. 2 Various surgical skills can be obtained through simulator training. 3 4

Femoroacetabular impingement (FAI) syndrome arises from abnormal contact between the rim of the acetabulum and the femoral neck during the range of motion, and the deformity most involved in this syndrome is the anomalous bulging in the anterosuperior portion of the femoral neck, known as the CAM. 5 The most common treatment currently used to correct this deformity is arthroscopic resection. Arthroscopic surgery is effective and correlated with good clinical results in various populations. 6 7 However, the most common cause of persistent symptoms and the need for revisions is incomplete resection of the deformity. 8 9 Additionally, there are reports in the literature that exaggerated or localized bone resection in inappropriate topographies, which would be related to persistent symptoms and risk of fractures. 10 11

Hip arthroscopy is known for having a steep learning curve, 12 and complications can be reduced with surgeons' experience. 13 Recently, several strategies have been implemented to increase the expertise of surgeons and decrease their difficulty in obtaining the skills needed to perform hip arthroscopies. 14

One of these is low-fidelity arthroscopic simulators, using models printed by additive manufacturing (3D printing). This study describes a realistic, low-fidelity arthroscopic simulator produced in Brazil with affordable materials. We also assess the ability of surgeons with different levels of experience to reproduce the desired correction.

## Methods

This is a comparative experimental study on realistic plastic models, three dimensional (3D) printed from medical images of a hip without bone deformities, in which surgeons' skills at different levels of experience in arthroscopic surgery for femoral CAM resection were evaluated.

A Digital Imaging and Communications in Medicine (DICOM) file stored in a medical image bank derived from a CT scan of a patient with a normal femur was obtained after consent and approval by the Research Ethics Committee.

**Fig. 1:**
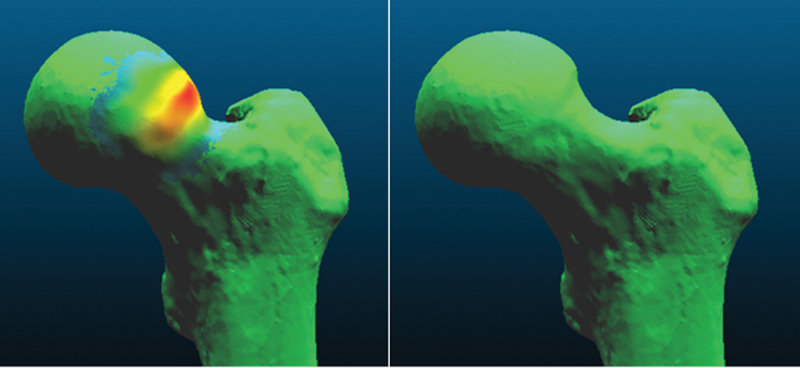
Images of femurs without (right) and with (left) deformity. Color highlight generated by the volumetric difference according to the CloudCompare software. **Source:** Photo by the author, December 2022.

The image of the patient's proximal femur was converted into a 3D mesh file (.stl) using the Invesalius software (Department of Computer Science at the Universidade Federal de Juiz de Fora). This file was called “femur without deformity.” The volume of the femur image without deformity was 128,073 mm ^3,^ and the surface area was 16,654.90 mm ^2^ . The image was then manipulated in the computer-aided design (CAD) program Meshmixer 2017 (Autodesk Inc., San Francisco, CA, USA), using extrusion and smoothing tools to create an image volumetrically like the typical deformity found in cam-type femoroacetabular impingement syndrome. This image was saved as “femur with deformity” ( Fig. 1 ). The volume of the deformed femur image was 130,601 mm ^3,^ and the surface area was 16,466.50 mm ^2^ . Therefore, the difference in volume between the femur with and without deformity was 1.988 mm ^3^ (i.e., 1.988 milliliters), and the difference in surface area was 188.4 mm ^2^ .

**Fig. 2:**
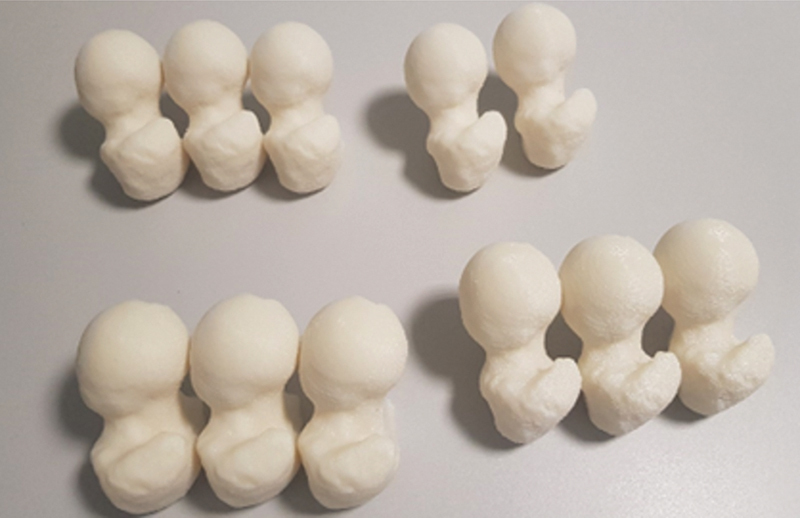
Femurs with deformities printed on ABS plastic. **Source:** Author, December 2022.

The files of the deformed femur were printed on a cast plastic injection printer using the rapid prototyping technique (3D printing) GTMax Core A1 (GTMax LTDA., Americana, SP, Brazil) ( Fig. 2 ).

### The Simulator

**Fig. 3:**
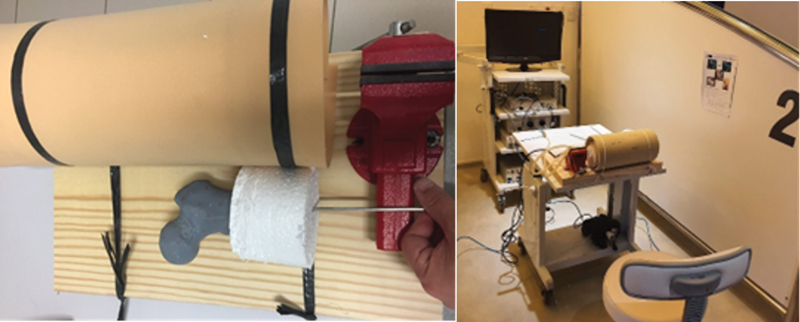
Elements of the simulator and assembly of the training station. Source: Author, January 2023.

The surgery was performed using a customized simulator created by the researchers. The simulator table consists of a flat wooden surface, a sergeant clamp, two PVC pipes (one 15 cm and the other 10 cm in diameter), two styrofoam cylinders (10 cm in diameter and 5 cm high), as well as medium-density foam (to simulate subcutaneous tissue) and a sheet of EVA (to simulate skin). To fix the plastic femurs in the simulator cavity, 5.5 mm Schanz pins were used ( Fig. 3 ).

### The Experiment

The 16 orthopedic doctors who had participated in an immersive hip arthroscopy course with practice on human cadavers, with different levels of experience in arthroscopic surgery, were invited to participate in the experiment, in which they performed simulated surgery to resect the deformity in plastic models. All participants agreed to take part, and there were no exclusions.

Participants answered a preparticipation questionnaire that recorded characteristics of their previous experience and degree of subspecialization.

Each participant was instructed on performing the procedure through a 30-minute lecture on the subject, with specific instructions on using the simulator. The simulated femoral osteoplasty procedure, using an arthroscopic simulation device, was carried out in a training room, using an arthroscopic camera with 70° optics and a 5.5mm arthroscopic bone resection blade (burr type). The researchers previously made two arthroscopic portals (anterolateral and distal medioanterior), and the instruments for performing the arthroscopic surgery were prepositioned. Each participant had 15 minutes to resect the deformity.

Immediately after the experiment, the participants answered a new questionnaire about their subjective perception of their abilities before and after the training and about their perceived experience of the simulator.

The specimens submitted to surgery on the simulator were optically scanned with a Desktop 3D Scanner HD (NextEngine, Santa Monica, CA, EUA) to assess the accuracy of the resection. The images from the optical scan were converted into stl-type digital files with 3D meshes and named “operated femur.”

The images of the operated femur were compared in the computer program CloudCompare (EDF S.A, Paris, França) v.2.10.2, with the images of the femur with and without deformity.

Comparisons made of the femur with deformity provided parameters on the volume of bone removed, speed of correction, and location of the correction. Comparisons of the femur without deformity were used to assess the ability to restore anatomy through bone resection (including the proportion of resection compared to what was expected and the presence or absence of overcorrection).

All comparisons were made by an evaluator blinded to the surgeons' experience.

We analyzed the maximum distance from the surface of the operated part to the one with and without deformity, the average absolute distance (includes only absolute values, not taking into account whether the surface is deeper or more externalized than the other), the average relative distance (contains both positive and negative values), the standard deviation (SD), the difference in volume and the total work done (obtained by subtracting the volume of the deformed part from the operated one and then dividing this number by the subtraction of the deformed part from the standard).

The 3D color maps provided images that allowed qualitative analysis of the resections.

## Results

### Comparison Between Deformed and Operated Femurs

The average difference in volume between the operated femurs and the femurs with deformities (resected volume) was of 0.52 ml (SD = 0.69 ml; max = 2.21; min = -0.04) ( Table 1 ).

**Table 1:** Metric and volumetric ratios between operated and deformed femurs

	Average	Deviation standard	Value Maximum	Value Minimum
**Maximum distance (mm)**	2,86	1,55	7,59	1
**Average absolute distance** **(mm)**	0,07	0,03	0,11	0,04
**Maximum error (mm)**	0,35	0	0,36	0,35
**Relative distance (mm)**	-0,03	0,04	0,03	-0,1
**Volumetric difference (ml)**	-0,52	0,69	-2,21	0,04

**Source:** Data generated by the author, based on comparisons in the CloudCompare software.

Proportionally, the average resection obtained was 26.2% (SD = 34.7%; max = 111; min = -2).

Data generated by the author from comparisons in the computer software CloudCompare.

Considering that the time available for the task was 15 minutes, the average resection speed was 34.66 mm ^3^ /minute (SD = 46 mm ^3^ /min, max = 147.33; min = -2.66). Therefore, the estimated time for resection of the entire deformity volume (if there were 100% accuracy) would be 57.35 minutes on average (SD = 43.21 min; min = 13.49; max = 747.36).

### Comparison of Non-deformed and Operated Femurs

The maximum distance between the operated and normal femurs, representing the residual deformity, was 5.15 mm on average (SD = 0.72 mm; max = 7.46; min = 4.54).

Regarding volume, the difference between operated femurs and those without deformity, which also expresses residual deformity, was of 1.9 ml (SD = 0.68 ml; max = 2.72; min = 0.26) ( Table 2 ).

**Table 2:** Metric and volumetric differences between operated and non-deformed femurs

	Average	Deviation Standard	Value Maximum	Value Minimum
**Maximum distance (mm)**	5,15	0,72	7,46	4,54
**Average absolute distance (mm)**	0,23	0,06	0,39	0,14
**Maximum error (mm)**	0,35	0	0,36	0,35
**Relative distance (mm)**	0,17	0,05	0,23	0,09
**Volumetric difference (ml)**	1,9	0,68	2,72	0,26

**Source:** Data generated by the author, based on comparisons in the CloudCompare software.

### Qualitative Analysis

**Fig. 4:**
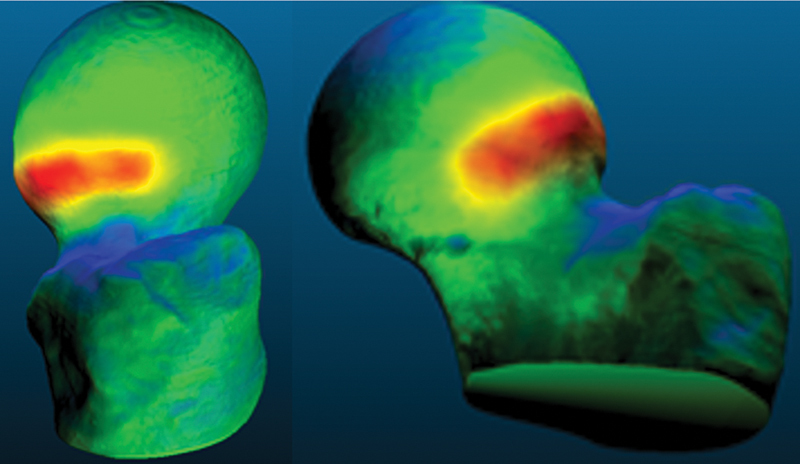
Example of insufficient correction in which the deformity (represented by the warm colors) has hardly been removed. **Source:** Author, based on comparisons in the CloudCompare software.

In addition to insufficient resection (average resection of 26.2%), qualitative analysis using 3D color maps showed a predominance of hypocorrection at the deformity site ( Fig. 4 ).

**Fig. 5:**
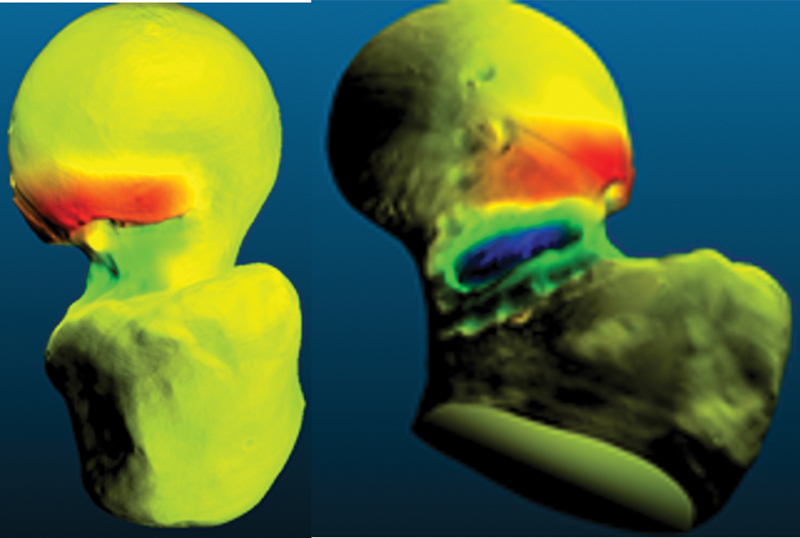
Case of inadequate resection shown by the excessively deep and distal area of bone resection (represented by the blue color) associated with the persistence of the deformity (represented by the red color). **Source:** Author, based on comparisons in the CloudCompare software.

Even in cases with a greater volume of correction, this occurred at the expense of resectioning areas unaffected by deformity to the detriment of the target areas with deformity ( Fig. 5 ).

### Answers to the Questionnaire ( Table 3 )

**Table 3:** Answers to the simulator evaluation questionnaire

	Disagree	Disagree	No	I agree	Agree, partially agree Agree, partially agree
	no disagree				
**The simulator is easy to use**	0	1(6,6%)	0	3(20%)	10(66,6%)
**The simulator reproduces the clinical condition from the FAI**	0	0	0	13(86,6%)	1(6,6%)
**The simulator requires the use of special arthroscopic skills**	0	0	2(12,12%)	8(53,3%)	3(20%)
**The simulator reproduces feeling tactile bone resection**	1(6,6%)	2(12,12%)	1(6,6%)	10(66,6%)	0
**The simulator is useful increasing surgical skills**	0	0	0	2(12,12%)	13(86,6%)
**The simulator could have been useful in the early stages of training**	0	0	0	1(6,6%)	13(86,6%)
**The simulator reproduces the spacial feeling of bone resection**	0	0	0	9(60%)	5(33,3%)
**This simulator should be part of the training of hip arthroscopists' in training**	0	0	0	0	14(93,3%)

**Abbreviation:** FAI, femoroacetabular impingement.

The study population included surgeons with different levels of previous experience: 20% were general orthopedic surgeons, 12.1% had a specialty in an area other than the hip, 6.6% were 1 ^st^ year residents, 6.6% were advanced hip surgeons (R4), and 53% were specialists in hip surgery according to the SBQ.

Concerning previous experience in arthroscopic surgery, 93.3% said they had never performed hip arthroscopy on human patients, and 93.3% had performed less than 30 arthroscopies in general. Concerning previous training, 60% said they had previously interacted with an arthroscopic simulator, and 40% had performed hip arthroscopies on human cadavers.

Regarding self-assessment of experience, 80% rated their level of expertise in arthroscopy as novice, 12% as intermediate, and 6% as advanced.

The users' perception of the simulator used in this study is of an easy to use tool (87.5%), reproducing the clinical deformity of the CAM in FAI (93.7%), which should be used as part of the training of hip arthroscopists in training (93.3%).

## Discussion

Femoroacetabular impingement repair surgery has many technical challenges, and various strategies have been proposed to overcome this steep learning curve. 15 16 Among surgeons, motor cognition skills are recognized as the most important for doctors in training to achieve competence. 14 Indeed, although training on cadavers seems to be the more favorable method for developing surgical skills, constraints of availability, time, and resources limit its frequent applicability. 14 Therefore, virtual reality simulators and high- and low-fidelity physical models have proved to be valuable alternatives for acquiring the complex motor skills required to learn hip arthroscopy. 14 16 The simulator presented in this study was developed in Brazil at an affordable cost and showed highly acceptable results among surgeons exposed to the training. In the literature, the most frequently reported parameters for evaluating the characteristics of simulator training were the ability to visualize and palpate joint structures with an arthroscopic probe (82%), average time to perform the task (73%), frequency of unwanted contact with cartilage and soft tissue (73%), and number of manual movements performed (73%). 16 In this study, we introduced additional objective parameters, namely the speed and volume of bone resection. The advantage of measuring these parameters is that they are directly related to the ability to accurately correct the deformity, which appears to be an essential element in the procedure's success in several studies.

Alter et al. 17 reported strong positive correlations between postoperative alpha angle measurements and the three-dimensional surface subtraction method, which is similar to the comparisons made in our study. In that study, the mean resected bone volume was 10,192 ± 486.2 mm ^3^ , which is close to the target resection values in our research and helps validate the verisimilitude of our model. Similarly, in that study, the maximum depth of bone resection measured was an average of 3.6 ± 1.0 mm, while in our series, we obtained values of 2.86 ± 1.55 mm. These values seem fully compatible, considering that, in our case, the participants only had 15 minutes to complete the task and, on average, only 26.2% of the expected volume was resected.

Inadequate resection of the deformity, whether in-depth, extent, or location, can increase the likelihood of postsurgical complications, including the risk of fractures and lip seal breakage. 10 11 An advantage of this simulator model is the possibility for the student to have instant feedback on the resection carried out, inspecting the operated part by simply removing it from the simulator after completing the task. Additionally, the quantitative and qualitative analysis carried out a posteriori with an optical scanner and computer programs can provide additional information to teachers and students in training.

Indeed, Uhl et al. 18 tested the use of 3D-printed simulators for cranioplasty training in patients with craniostenosis and found a significant improvement in the doctors' technique. In this study, 93.3% of the participants reported that they would have liked to have had access to the simulator at an earlier stage in their medical training, in addition to responding that training with the simulator should be applied while teaching the arthroscopic technique. The same observation was made by most of the surgeons involved in our study. Karam et al., 19 in a survey carried out in conjunction with the American Academy of Orthopedic Surgeons, found that 80% of orthopedic residency directors believe that the use of surgical simulations is essential for training residents in basic skills, and 87% say that the most significant barrier to instituting this training for their students is the lack of funds to pay for the simulators' high cost. Regarding residents, 66% say there is a need to implement a laboratory in all residencies in the country. 4

The present study has several limitations. The lack of a control group with expert surgeons limits the validation of our model. However, as it was a pilot study, it made estimating the time allocated to complete the task (up to 60 min) possible. It also allowed us to identify the potential of the optical scanning method associated with 3D printing as a research and teaching tool. Printing errors are reported in the literature and can account for up to 1.1% of printed volumes. 20 This explains the negative values found in some linear and volumetric comparisons. Although this may impact the reliability of the measurements, this limitation does not affect the qualitative analysis or prevent the simulator's application. In future research, it would be advisable to scan the printed part optically before the procedure rather than compare it to the original digital model. This measure would eliminate potential bias due to dimensional printing errors.

## Conclusion

Arthroscopic simulators produced by 3D printing are helpful in training surgeons to resect cam deformities in hip arthroscopy. The average speed of CAM deformity resection in this study was 34.66 mm ^3^ /minute, and the estimated time for complete resection was of 57.35 minutes.
