# Supplementary material for: Analysis of the Accuracy of CAM-type Deformity Resection on a Low-cost Arthroscopic Simulator in a Training Scenario
Source: Rev Bras Ortop (Sao Paulo). 2024 Jun 22;59(3):e449–55. doi: 10.1055/s-0044-1785666 (PMC11193573; doi:10.1055/s-0044-1785666)
Supplement: Related texts

# Research article: Análise da acurácia da ressecção de deformidades tipo CAME em simulador artroscópico de baixo custo em um cenário de treinamento

**Keywords:** artroscopia, impacto femoroacetabular, quadril, treinamento por simulação

## Abstract

**Objetivo** Avaliar o desempenho de cirurgiões na ressecção de deformidades tipo CAME fazendo uso de um simulador realista de cirurgia artroscópica.

**Métodos** Um simulador artroscópico foi criado a partir de materiais de baixo custo com auxílio de uma impressora 3D GTMax Core A1 e dos programas Invesalius e Meshmixer 2017, que foram utilizados para criar peças de cabeça de fêmur em material ABS, com a presença de uma deformidade tipo CAME, a fim de mimetizar uma situação de impacto femoroacetabular. Após as operações serem realizadas por 16 cirurgiões, os fêmures foram comparados a um modelo prévio com e outro sem deformidade pelo programa Cloudcompare, sendo avaliados ao final parâmetros como diferença volumétrica entre os fêmures operados, com e sem deformidade, distância mínima e máxima entre eles, porcentagem da deformidade ressecada, tempo estimado para ressecção total da deformidade, além de uma análise qualitativa feita com base nas imagens e gráficos fornecidos pelo programa representando as áreas das peças efetivamente ressecadas.

**Resultados** A velocidade média de resseção foi de 34,66mm ^3^ /minuto (DP = 46 mm ^3^ /min, max = 147,33 mm ^3^ /min; min = -2,66 mm ^3^ /min). A média de ressecção obtida foi de 26,2% (DP = 34,7%, max = 111%; min = -2%). A análise qualitativa demonstrou uma hiporresecção das deformidades e, por vezes, hiperresecção de áreas não deformadas. O simulador foi muito bem avaliado pelos cirurgiões, tendo uma sensação tátil bem semelhante à cirurgia real segundo os mesmos.

**Conclusão** Simuladores artroscópicos se mostraram muito úteis no treinamento de cirurgiões menos experientes.

## Introdução

O treinamento de cirurgiões para aquisição de habilidade motoras complexas é um fator decisivo no resultado das cirurgias ortopédicas. Em âmbito hospitalar o maior volume de casos tem sido fortemente associado com a melhora do tempo de recuperação no pós-operatório, diminuição de mortalidade e custos. 1 O volume de treinamento gera aumento da eficiência de cirurgiões no centro cirúrgico e da segurança do paciente. 2 Diversas habilidades cirúrgicas podem ser obtidas por meio de treinamento em simuladores. 3 4

A síndrome do impacto femoroacetabular (SIFA) decorre de um contato anormal entre o rebordo do acetábulo e o colo do fêmur, durante o arco de movimento, e a deformidade mais comumente envolvida nesta síndrome é o abaulamento anômalo na porção anterossuperior do colo do fêmur, denominada de CAME. 5 O tratamento mais utilizado atualmente para corrigir esta deformidade é a ressecção artroscópica. A cirurgia artroscópica é eficaz e correlacionada a bons resultados clínicos em diversas populações. 6 7 No entanto, a causa mais comum de persistência dos sintomas e necessidade de revisões é a ressecção incompleta da deformidade. 8 9 Além disso, há relatos na literatura de ressecção óssea exagerada ou localizada em topografias inadequadas que estariam relacionadas a sintomas persistentes e risco fraturas. 10 11

De fato, a artroscopia de quadril é reconhecida por possuir uma íngreme curva de aprendizado 12 e as complicações podem ser diminuídas com a experiência do cirurgião. 13 Recentemente, várias estratégias têm sido implementadas para aumentar a expertise de cirurgiões e diminuir sua dificuldade em obter as habilidades necessárias para a realização de artroscopias de quadril. 14

Uma delas é a utilização de simuladores artroscópicos de baixa fidelidade, utilizando modelos impressos em por manufatura aditiva (impressão 3D). Neste estudo descrevemos um simulador artroscópico realístico, de baixa fidelidade, produzido no Brasil com materiais de custo acessível, e avaliamos a capacidade de cirurgiões com diversos níveis de experiência em reproduzir a correção desejada.

## Método

Trata-se de um estudo experimental comparativo em modelos plásticos realísticos, impressos em três dimensões (3D) a partir de imagens médicas de um quadril sem deformidades ósseas; no qual a habilidade de cirurgiões em diferentes níveis de experiência em cirurgia artroscopia de ressecção do CAME femoral foram avaliadas.

Um arquivo Digital Imaging and Communications in Medicine (DICOM) armazenado em um banco de imagens médicas derivado de tomografia de um paciente com fêmur normal, foi obtido após consentimento e aprovação pelo Comitê de Ética em Pesquisa.

A imagem do fêmur proximal do paciente foi convertida em arquivo de malhas tridimensionais (.stl) utilizando o software Invesalius (Departamento de Ciências da Computação da Universidade Federal de Juiz de Fora). Este arquivo foi denominado “fêmur sem deformidade”. O volume da imagem do fêmur sem deformidade foi de 128.073 mm ^3^ e a área de superfície de 16.654,90 mm ^2^ . A imagem foi então manipulada no programa de desenho assistido por computador (CAD) Meshmixer 2017 (Autodesk Inc., São Francisco, CA, EUA), utilizando ferramentas de extrusão e suavização, de forma a criar uma imagem volumetricamente semelhante a deformidade típica encontrada na síndrome do impacto femoroacetabular tipo CAME. Esta imagem foi salva como “fêmur com deformidade” ( Fig. 1 ). O volume da imagem do fêmur com deformidade foi de 130.601 mm ^3^ e a área de superfície de 16.466,50 mm ^2^ . Portanto, a diferença de volume entre o fêmur com e sem deformidade foi de 1.988 mm ^3^ (ou seja 1,988 mililitros) e a diferença de área de superfície de 188,4 mm ^2^ .

**Fig. 1:** Imagens dos fêmures sem (direita) e com (esquerda) deformidade. Destaque de cores gerados pela diferença volumétrica conforme no programa CloudCompare. **Fonte:** Autor, dezembro de 2022.

Os arquivos do fêmur com deformidade foram impressos em com impressora injetora de plástico fundido por técnica de prototipagem rápida (impressão 3D) GTMax Core A1 (GTMax LTDA., Americana, SP, Brasil) ( Fig. 2 ).

**Fig. 2:** Fêmures com deformidades impressos em plástico ABS. **Fonte:** Autor, dezembro de 2022.

## O Simulador

A cirurgia foi realizada por intermédio de um simulador customizado criado pelos próprios pesquisadores. A mesa simuladora consiste em uma superfície plana de madeira, um grampo sargento, dois canos de PVC (um com 15 e outro com 10 cm de diâmetro), dois cilindros de isopor (com 10 cm de diâmetro e 5 cm de altura), além de espuma de média densidade (para simular o tecido subcutâneo) e uma folha de EVA (para simular a pele). Para a fixação dos fêmures plásticos na cavidade do simulador foram utilizados pinos de Schanz de 5.5mm ( Fig. 3 ).

**Fig. 3:** Elementos do simulador e montagem da estação de treinamento. **Fonte:** Autor, janeiro de 2023.

## O Experimento

Os 16 médicos ortopedistas que participaram de curso imersivo de artroscopia de quadril com prática em cadáveres humanos, com diferentes níveis de experiência em cirurgia artroscópica, foram convidados a participar do experimento, no qual realizaram uma cirurgia simulada de ressecção da deformidade nos modelos plásticos. Todos os participantes aceitaram participar e não houve exclusões.

Os participantes responderam a um questionário preparticipação que registrou características sobre sua experiencia prévia e grau de subespecializarão.

Cada participante recebeu instrução para realização do procedimento por meio de aula de 30 minutos a respeito do tema, com instruções específicas de como utilizar o simulador. O procedimento simulado de osteoplastia femoral, em dispositivo de simulação artroscópica foram executados em uma sala de treinamento, com auxílio de câmera artroscópica e ótica de 70° e lâmina de ressecção artroscópica óssea (tipo burr) de 5.5mm. Dois portais artroscópicos (anterolateral e medioanterior distal) foram previamente feitos pelos pesquisadores e os instrumentais para a realização da cirurgia artroscópica foram preposicionados. Cada participante teve 15 minutos para realizar a ressecção da deformidade.

Imediatamente após o experimento, os participantes responderam a um novo questionário sobre a sua percepção subjetiva de suas capacidades antes e após o treinamento, bem como sobre sua percepção da experiência do simulador.

Para avaliação da acurácia da ressecção as peças submetidas a cirurgia no simulador foram escaneadas opticamente pela Desktop 3D Scanner HD (NextEngine, Santa Monica, CA, EUA). As imagens obtidas no escaneamento óptico foram convertidas em arquivos digitais formato stl com malhas 3D e nomeadas como “fêmur operado”.

As imagens do fêmur operado foram comparadas no programa de computador CloudCompare (EDF S.A, Paris, França) v.2.10.2, com as imagens do fêmur com e sem deformidade.

As comparações do fêmur com deformidade forneceram parâmetros sobre o volume de osso removido, velocidade de correção e localização da correção. As comparações do fêmur sem deformidade serviram para avaliar a capacidade de restauração da anatomia por meio da ressecção óssea (incluindo a proporção de ressecção em relação ao esperado e a presença ou não de hipercorreção).

Todas as comparações foram feitas por um avaliador cego para a experiência dos cirurgiões.

Foram analisadas a distância máxima da superfície da peça operada para a com e sem deformidade, distância média absoluta (engloba somente valores absolutos, não levando em conta se a superfície está mais profunda ou externalizada do que a outra), média de distância relativa (engloba tanto valores positivos quanto negativos), desvio padrão (DP), diferença de volume e trabalho total realizado (obtido a partir da subtração do volume da peça deformada pela operada e então a divisão desse número pela subtração da peça deformada para a norma).

Os mapas 3D coloridos forneceram imagens que permitem a análise qualitativa das resseções e são apresentados.

## Resultados

## Comparação Entre os Fêmures com Deformidade e Operados

A média da diferença de volume entre os fêmures operados e os fêmures com deformidade (volume ressecado) foi de 0,52 ml (DP = 0,69 ml, max = 2,21; min = -0,04) ( Tabela 1 ).

**Tabela 1:** Relações métricas e volumétricas entre os fêmures operados e com deformidade

	Média	DP	Valor Máximo	Valor Mínimo
**Distância máxima (mm)**	2,86	1,55	7,59	1
**Distância média absoluta** **(mm)**	0,07	0,03	0,11	0,04
**Erro máximo (mm)**	0,35	0	0,36	0,35
**Distância relativa (mm)**	-0,03	0,04	0,03	-0,1
**Diferença volumétrica (ml)**	-0,52	0,69	-2,21	0,04

**Abreviações:** DP, desvio padrão. **Fonte:** Dados gerados pelo autor, a partir de comparações no programa de computador CloudCompare.

Proporcionalmente a média de ressecção obtida foi de 26,2% (DP = 34,7%, max = 111%; min = -2%).

Dados gerados pelo autor, a partir de comparações no programa de computador CloudCompare.

Considerando o tempo disponível para a tarefa de 15 minutos, a velocidade média de resseção foi de 34,66mm ^3^ /minuto (DP = 46 mm ^3^ /min; max = 147,33 mm ^3^ /min; min = -2,66 mm ^3^ /min). Assim sendo, o tempo estimado para uma ressecção do volume completo da deformidade (caso houvesse 100% de acurácia) seria de 57,35 minutos em média (DP = 43,21 min; min = 13,49 min; max = 747,36 min).

## Comparação Entre Fêmures Sem Deformidade e Operados

A distância máxima entre os fêmures operados e normais, que representa a deformidade residual, foi em média 5,15 mm (DP = 0,72 mm; max = 7,46 mm; min = 4,54 mm).

Em termos de volume, a diferença entre os fêmures operados e aqueles sem deformidade, que também expressa deformidade residual, foi de 1,9 ml (DP = 0,68 ml; max = 2,72 ml; min = 0,26 ml) ( Tabela 2 ).

**Tabela 2:** Diferenças métricas e volumétricas entre fêmures operados e sem deformidade

	Média	DP	Valor Máximo	Valor Mínimo
**Distância máxima (mm)**	5,15	0,72	7,46	4,54
**Distância média absoluta (mm)**	0,23	0,06	0,39	0,14
**Erro máximo (mm)**	0,35	0	0,36	0,35
**Distância relativa (mm)**	0,17	0,05	0,23	0,09
**Diferença volumétrica (ml)**	1,9	0,68	2,72	0,26

**Abreviações:** DP, desvio padrão. **Fonte:** Dados gerados pelo autor, a partir de comparações no programa de computador CloudCompare.

## Análise Qualitativa

Além de uma ressecção insuficiente (média de ressecção de 26,2%), a análise qualitativa por estudo dos mapas de cores 3D evidenciou um predomínio de hipocorreção no sítio da deformidade ( Fig. 4 ).

**Fig. 4:** Exemplo de correção insuficiente em que a deformidade (representada pelas cores quentes) praticamente não foi removida. **Fonte:** Autor, a partir de comparações no programa de computador CloudCompare.

Mesmo nos casos em que houve maior volume de correção, este ocorreu às custas de resseção de áreas não acometidas por deformidade em detrimento das áreas alvo com deformidade ( Fig. 5 ).

**Fig. 5:** Caso de ressecção inadequada demonstrada pela área demasiadamente profunda e distal da ressecção óssea (representada pela cor azul) associada a persistência da deformidade (representada pela cor vermelha). **Fonte:** Autor, a partir de comparações no programa de computador CloudCompare.

## Respostas ao Questionário ( Tabela 3 )

**Tabela 3:** Respostas ao questionário de avaliação do simulador

	Discordo	Discordo	Não	Concordo	Concordo totalmente parcialmente Concordo, parcialmente totalmente
	não discordo				
**O simulador é de fácil utilização**	0	1(6,6%)	0	3(20%)	10(66,6%)
**O simulador reproduz a** **condição clínica do IFA**	0	0	0	13(86,6%)	1(6,6%)
**O simulador necessita do uso de habilidades artroscópicas especiais**	0	0	2(12,12%)	8(53,3%)	3(20%)
**O simulador reproduz sensação** **tátil da ressecção óssea**	1(6,6%)	2(12,12%)	1(6,6%)	10(66,6%)	0
**O simulador é útil em aumentar as habilidades cirúrgicas**	0	0	0	2(12,12%)	13(86,6%)
**O simulador poderia ter sido útil em fases iniciais do treinamento**	0	0	0	1(6,6%)	13(86,6%)
**O simulador reproduz a sensação espacial da ressecção óssea**	0	0	0	9(60%)	5(33,3%)
**Esse simulador deveria ser parte do treinamento de artroscopistas de quadril em formação**	0	0	0	0	14(93,3%)

A população do estudo incluiu cirurgiões com diferentes níveis de experiência prévia, sendo 20% ortopedista generalistas, 12,1% com especialidade em área distinta ao quadril, 6,6 % residentes do 1° ano, 6,6% em nível de aperfeiçoamento em cirurgia de quadril (R4) e 53% especialistas em cirurgia de quadril pela SBQ.

Em relação à experiência prévia em cirurgias artroscópicas, 93,3% responderam nunca terem realizado artroscopia de quadril em pacientes humanos e 93,3% realizaram menos de 30 artroscopias no geral. Em relação a treinamentos prévios, 60% afirmaram já terem interagido com algum simulador artroscópico e 40% já realizaram artroscopias de quadril em cadáveres humanos.

Quanto a autoavaliação da experiência, 80% qualificaram seu nível de experiência em artroscopia como novatos, 12% como intermediários e 6% como avançados.

A percepção dos usuários a respeito do simulador utilizado nesta pesquisa é de uma ferramenta fácil utilização (87,5%), reproduzindo a deformidade clínica do CAME na SIFA (93,7%), devendo ser utilizado como parte do treinamento de artroscopistas de quadril em treinamento (93.3%).

## Discussão

A cirurgia de correção do impacto femoroacetabular é cercada de desafios técnicos e diversas estratégias têm sido propostas para vencer esta íngreme curva de aprendizado. 15 16 Entre os cirurgiões, as habilidades de cognição motoras são reconhecidas como as mais importantes para que o médico em treinamento atinja a competência. 14 De fato, embora o treinamento em cadáveres pareça ser o método mais favorável ao desenvolvimento das habilidades cirúrgicas, restrições de disponibilidade, tempo e recursos limitam sua aplicabilidade frequente. 14 Desta forma, simuladores de realidade virtual e modelos físicos de alta e baixa fidelidade têm se mostrado alternativas úteis na aquisição das habilidades motoras complexas necessárias para o aprendizado da artroscopia de quadril. 14 16 O simulador apresentado nesta pesquisa foi desenvolvido no Brasil, a um custo acessível e apresentou resultados de alta aceitação entre os cirurgiões expostos ao treinamento. Na literatura, os parâmetros mais frequentemente reportados para avaliar as características do treinamento em simuladores foram a habilidade de obter visualização e palpação de estruturas articulares com sonda artroscópica (82%), tempo médio para realizar a tarefa (73%), frequência de contatos indesejados com cartilagem e tecidos moles (73%), e quantidade de movimentos manuais realizados (73%). 16 Nesta pesquisa, introduzimos parâmetros objetivos adicionais que são a velocidade e o volume de ressecção óssea. A vantagem de mensurar estes parâmetros é que eles se relacionam diretamente com a habilidade de obter uma correção acurada da deformidade, que parece ser um elemento essencial para o sucesso do procedimento em vários estudos.

Alter et al. 17 relataram fortes correlações positivas entre as medidas de ângulo alfa pós-operatórias e o método de subtração de superfícies tridimensionais, que é semelhante as comparações realizadas no nosso estudo. Naquela pesquisa, as médias de volume ósseo ressecado foram de 10.192 ± 486,2 mm ^3^ , o que se aproxima dos valores alvo de ressecção em nossa pesquisa a ajudam a validar a verossimilhança do nosso modelo. Da mesma forma, naquele estudo, a profundidade máxima de resseção óssea medida foi em média de 3.6 ± 1.0 mm, enquanto em nossa casuística obtivemos valores de 2,86 ± 1,55 mm. Estes valores parecem plenamente compatíveis, considerando que, em nosso caso, os participantes tiveram apenas 15 minutos para executar a tarefa e, em média, apenas 26,2% do volume esperado foi ressecado.

A ressecção inadequada da deformidade, seja em profundidade, extensão e localização podem aumentar probabilidade de complicações pós-cirúrgicas, incluindo o risco de fraturas e quebra do selo labial. 10 11 Uma vantagem deste modelo de simulador é a possibilidade de o aluno ter o feedback instantâneo da ressecção realizada, inspecionando a peça operada, simplesmente retirando-a do simulador após o término da tarefa. Adicionalmente, a análise quantitativa e qualitativa, realizada a posteriori com auxílio de escâner óptico e programas de computador, pode fornecer informações adicionais aos professores e alunos em treinamento.

De fato, Uhl et al. 18 testaram o uso de simuladores feitos com impressão 3D para treinamento de cranioplastia em pacientes com cranioestenose e encontrou melhora significativamente na técnica dos médicos que usufruíram do simulador. Neste trabalho, 93,3% dos participantes relataram que gostariam de ter acesso ao simulador em estágios mais precoces de sua formação médica, além de responderem que o treinamento com o simulador deveria ser aplicado durante o ensino da técnica artroscópica. A mesma observação foi feita pela maioria dos cirurgiões envolvidos na nossa pesquisa. Karam et al., 19 em pesquisa feita em conjunto com a academia americana de cirurgiões ortopédicos, identificaram que 80% dos diretores de residência em ortopedia acreditam que o uso de simulações cirúrgicas é essencial para o treinamento de habilidades básicas para os residentes e 87% dizem que a maior barreira para instituir esse treinamento para seus alunos é a falta de fundos para arcar com os altos custos dos simuladores. Já com relação aos residentes, 66% deles apontam a necessidade da implementação de laboratório em todas as residências do país. 4

O presente estudo tem várias limitações. A falta de um grupo controle com cirurgiões especialistas limita a validação do nosso modelo. Não obstante, por ser um estudo piloto, possibilitou estimar o tempo a ser designado para a realização da tarefa completa (até 60 min). Além disso, foi possível identificar o potencial do método de escaneamento ótico associada a impressão 3D como ferramenta de pesquisa e didática. Os erros de impressão são relatados na literatura e podem responder por até 1,1% dos volumes impressos. 20 Isso explica os valores negativos encontrados em algumas comparações lineares e volumétricas. Embora possa tem impacto negativo na confiabilidade das medidas, essa limitação não afeta a análise qualitativa nem impede a aplicação do simulador. Em pesquisas futuras, convém realizar o escaneamento ótico da peça impressa antes do procedimento, ao invés de comparar com o modelo digital original. Essa medida eliminaria o potencial viés, decorrente dos erros dimensionais de impressão.

## Conclusão

Simuladores artroscópicos produzidos por impressão 3D são úteis no treinamento de cirurgiões para ressecção da deformidade tipo CAME na artroscopia de quadril. A velocidade média de resseção neste estudo foi de 34,66mm ^3^ /minuto e o tempo estimado para uma ressecção completa foi de 57,35 minutos.
